# Differentiate Responses of Soil Microbial Community and Enzyme Activities to Nitrogen and Phosphorus Addition Rates in an Alpine Meadow

**DOI:** 10.3389/fpls.2022.829381

**Published:** 2022-03-02

**Authors:** Hongbiao Zi, Lei Hu, Changting Wang

**Affiliations:** ^1^State Key Laboratory of Grassland Agro-Ecosystems, and College of Pastoral Agriculture Science and Technology, Lanzhou University, Lanzhou, China; ^2^Institute of Qinghai-Tibetan Plateau Research, Southwest Minzu University, Chengdu, China

**Keywords:** N and P addition, phospholipid fatty acid, soil enzyme activities, rate dependent, alpine meadow

## Abstract

Nitrogen (N) and phosphorus (P) are the dominant limiting nutrients in alpine meadows, but it is relatively unclear how they affect the soil microbial community and whether their effects are rate dependent. Here, N and P addition rates (0, 10, 20, and 30 g m^–2^ year^–1^) were evaluated in an alpine meadow and variables related to plants and soils were measured to determine the processes affecting soil microbial community and enzyme activities. Our results showed that soil microbial biomass, including bacteria, fungi, gramme-negative bacteria, and actinomycetes, decreased along with N addition rates, but they first decreased at low P addition rates (10 g m^–2^ year^–1^) and then significantly increased at high P addition rates (30 g m^–2^ year^–1^). Both the N and P addition stimulated soil invertase activity, while urease and phosphatase activities were inhibited at low N addition rate and then increased at high N addition rate. P addition generally inhibited peroxidase and urease activities, but increased phosphatase activity. N addition decreased soil pH and, thus, inhibited soil microbial microorganisms, while P addition effects were unimodal with addition rates, achieved through altering sedge, and available P in the soil. In conclusion, our studies indicated that soil microbial communities and enzyme activities are sensitive to short-term N and P addition and are also significantly influenced by their addition rates.

## Introduction

Nitrogen (N) and phosphorus (P) addition, either individually or in combination, have been shown to influence the above- and belowground ecosystem performance in many terrestrial ecosystems (Humbert et al., 2016; Li et al., 2016). N and P fertiliser addition increased plant biomass relative to unfertilised plants by 81 and 22%, respectively (Craine and Jackson, 2009), but reduced plant community richness and diversity (Chen et al., 2013; Isbell et al., 2013). In addition, a marked difference showed that N addition favoured grasses (Clark and Tilman, 2008), while P addition favoured legume growth (Zhao et al., 2019) due to N fixing (Marleau et al., 2011).

As an important component of the belowground ecosystems, soil microorganisms usually interact with the plants through roots and, thus, form cooperative or competitive relationships (Zhou et al., 2021). In most ecosystems, plants would supply labile carbon and receive nutrients from microorganisms in return (Brundrett, 2009). In some cases, soil microorganisms are strong competitors for N or P, at least in the short term, because they temporarily incorporate limiting nutrients in their biomass, making them unavailable to nutrient-limited plants (Kuzyakov and Xu, 2013; Wild et al., 2018). In general, plant diversity and density are directly correlated with soil microbial diversity and function (Porazinska et al., 2018; Navratilova et al., 2019) and soil microbial biomass also directly depends on plant species richness (Geisseler and Scow, 2014). These plant effects on soil microorganisms indicated that changes in plants would surely affect soil microbial community, while these effects remain elusive when N or P fertilisers are applied.

Except for plant effects, fertiliser addition directly alters soil microbial community, especially depending on addition rate. A meta-analysis showed that N addition could inhibit soil microbial diversity and 15% of soil microbial biomass on average (Wang et al., 2018) for significant changes in soil pH (Lucas et al., 2011; Wang et al., 2017), but a more copiotrophic microbial phyla or fungal biomass would increase (Cusack et al., 2011; Fierer et al., 2012). However, other studies demonstrated that N addition had neutral or slightly negative effects on soil microbial abundance and activity (Treseder, 2004; Camenzind et al., 2018). The soil microbial responses to N addition also varied between ecosystem types. In a semi-arid temperate steppe, soil microbial biomass slightly increased at low N addition rate, but decreased at high N addition rate (Zhang et al., 2008). In an alpine meadow, soil microorganisms, such as the arbuscular mycorrhizal fungal community, were influenced by N addition and the high N rate decreased the arbuscular mycorrhizal fungal spore (Zheng et al., 2014). In a subtropical agricultural ecosystem, organic fertilisers would also improve soil microbial activity, but microbial growth would not increase with fertiliser application (Dong et al., 2014). However, in a lowland tropical rain forest, the response of soil microbial biomass to a decade of N addition was not significant (Turner and Joseph, 2013). N-rich tropical forests even have an increase in fungal biomass with N addition (Cusack et al., 2011). The different soil microorganism responses showed us that the effect of N addition might depend on the interaction between the studied ecosystem types and the N addition rate (Geisseler and Scow, 2014; Yao et al., 2014; Zhou et al., 2016).

Unlike N addition effects, P addition effects on soil microorganisms are relatively unknown (Treseder, 2008), especially in alpine meadows. Soil pH, the key factor influencing soil microorganisms, was not significantly changed by P addition (Mao et al., 2017) and it did not even increase, as shown by a meta-analysis (Xiao et al., 2018). However, P addition directly alters the plant community productivity, especially the legume biomass (Zhao et al., 2019), and it indirectly significantly affects the surrounding microorganisms (Hartmann et al., 2008; Berendsen et al., 2012) through rhizodeposition (Zak et al., 2003; Eisenhauer et al., 2010) or symbiotic N fixation (Augusto et al., 2013; Diáková et al., 2018) and the increasing mycorrhizal colonisation of plant roots (Lagrange et al., 2013). Thus, the effects of P addition on soil microorganisms might be correlated with plant factors, but not with soil factors.

Otherwise, the effects of N and P addition interact and directly change the soil nutrient ratio in many ecosystems. With increasing N addition, the limiting effect of P on the aboveground net primary production would increase severely (Li et al., 2016). P addition would also augment the effects of N addition on grassland such as soil respiration (Liu et al., 2020). The alpine meadow, the dominant grassland type of the Qinghai-Tibet Plateau, has been also demonstrated to be N and P colimited (Xu et al., 2015; He et al., 2016). Most studies conducted in alpine meadows were only focussed the effects of N addition on plant or soil microbial community characteristics (Ren et al., 2016; Guo et al., 2017; Wang et al., 2017, 2019). Some studies have demonstrated a neutral effect of N addition, but a negative effect of P addition on extracellular enzymatic activities (Jing et al., 2016), where P was the key limiting factor for soil microorganisms in alpine meadows (Dong et al., 2020). Overall, few studies simultaneously have showed how N and P affect the soil microbial community and whether their effects are rate dependent.

Here, N and P addition was conducted in alpine meadows and the plant community, soil microbial community, as well as soil physicochemical properties were measured. We first aimed to differentiate the effects of N and P addition on soil microbial community and then we focussed on the extent of their effect by evaluating N and P addition rates in an alpine meadow in the Qinghai-Tibet Plateau. We aimed to: (1) determine that the responses of soil microbial communities and enzyme activities to N and P addition rates and (2) explore the main abiotic and biotic factors structuring soil microbial community. Based on the previous studies, we hypothesised that: (1) the effect of N and P addition on the soil microbial community would be rate dependent, especially in the different soil microbial groups and (2) different soil and plant factors drive soil microorganisms under N and P addition.

## Materials and Methods

### Study Site

This experiment was conducted in an alpine meadow, located at Hongyuan County (N: 32°50′–33°22′, E: 102°01′–103°23′) of the Qinghai-Tibet Plateau. With an average altitude of 3,500 m, the Hongyuan alpine meadow has a mean annual temperature of 0.9°C ranging from −10.3 at January to 10.9°C at July and mean annual precipitation of 657–730 mm, characterised by the high altitude and low temperature all the year round. Based on the field survey, the natural plant community was dominated by Cyperaceae plants such as *Kobresia humilis* and *Blysmus sinocom*, Gramineae plants such as *Elymus nutans* and *Agrostis clavata*, accompanying by Compositae plants such as *Saussurea nigrescens* and *Anaphalis lacteal* and Ranunculaceae plants such as *Anemone trullifolia* and *Anemone rivularis*. The soil at this site was Mollisols, with low pH (4.6–6.0) and soil organic matter (SOM) ranging from 80 to 120 g⋅kg^–1^ (Hu et al., 2017).

### Experimental Design

The two factors, N addition (four addition rates: CK, 0; N10, 10 g m^–2^ year^–1^, N20, 20 g m^–2^ year^–1^, and N30, 30 g m^–2^ year^–1^) and P addition (four addition rates: CK, 0; P10, 10 g m^–2^ year^–1^, P20, 20 g m^–2^ year^–1^, and P30, 30 g m^–2^ year^–1^), in a completely randomised block design, formed 7 treatments with 6 replications. There were 42 subplots (3 m × 3 m) separated by 2-m buffer zones to avoid the marginal effect located in the 300 m × 300 m alpine meadow site fenced with wire netting to prevent disturbance from grazing or other animals. The N fertiliser of urea [CO(NH_2_)_2_] and P fertiliser of calcium superphosphate [Ca(H_2_PO_4_)⋅H_2_O] were uniformly added once a year in a cloudy day of early growing seasons in the alpine meadow since late April 2012.

### Field Sampling

On three randomly selected subplots from the six ones, all the plants within a 50 cm × 50 cm quadrat were harvested and separated into four functional groups (grasses, sedges, legumes, and forbs) in the late August 2013. For each subplot, the height, abundance, and coverage of each species recorded.

After removal of aboveground plants, soil samples in 0–10 cm were randomly collected by a soil auger (5 cm in diameter) in a V-shaped pattern for 5 times from the selected subplots. Soil samples were uniformity mixed and then transported in cooled boxes to the laboratory and sieved (0.25 mm). One part was stored at 4°C for measuring soil microbes and soil enzyme activities and the others were dried for soil pH and soil nutrients. Fine roots were sampled in five soil cores (5 cm in diameter) at 0–10 cm and then isolated by sieving 2 mm mesh. The total roots and the mowed aboveground plants were dried at 60°C for 48 h and then weighed.

### Soil Physicochemical Properties

The air-dried soil samples were sieved through a 2-mm mesh to measure their physicochemical properties. Soil pH was determined with a glass electrode by using a soil to water ratio of 1:1 (Chu et al., 2007). SOM concentration was determined by a modification of the chromic acid titration method—Degtjareff method (Walkley and Black, 1934). Soil total N (TN) was determined by the Kjeldahl method (Bremner, 1960). Soil total P (TP) was determined by after digestion in HF-HClO_4_ (Jackson, 1974). The available N (AN) was measured by using an automated procedure (Skalar SANplus Segmented Flow Analyser, Skaler Incorporation, Breda, Netherlands) and the available P (AP) was assayed by molybdenum blue colorimetry and flame photometry and measured with a spectrophotometer (Hach DR 2700, Hach Company, Loveland, Colorado, United States).

### Soil Microbial Community and Enzyme Activities

Soil microbial communities were assessed by using phospholipid fatty acid (PLFA) analysis described by Frostegård et al. (1993). Based on the previous results and the method of PLFA biomarkers, the appraised carbon chain length of measured PLFA was C13–C18 for abundant species, including saturated, unsaturated, and cyclic PLFAs, of which there were 24 types in total. The Gramme-positive bacteria (G^+^) were estimated as the sum of i15:0, i16:0, i17:0, i18:0, a15:0, a17:0, and a18:0 and the Gramme-negative bacteria (G^–^) were estimated as the sum of cy15:0, cy17:0, 16:1ω7c, 16:1ω9c, 18:1ω7t, and 18:1ω7c (Frostegård et al., 1993; Frostegård and Bååth, 1996; McKinley et al., 2005; Ramsey et al., 2006). Actinomycetes (Ac) were estimated as the sum of 2Me 18:0, 10Me 17:0, and 10Me 18:0 (McKinley et al., 2005) and fungi were estimated as the sum of 18:1ω9c and 18:2ω69c (Frostegård and Bååth, 1996; Frostegård et al., 2011). The left PLFAs were indicative of general bacteria (GB) (Frostegård et al., 1993; Frostegård and Bååth, 1996).

The substrate status of soil microorganisms could be inferred from measurements of soil enzyme activities because investment in enzyme synthesis was assumed to reflect biological nutrient demand (Turner and Joseph, 2013). Soil enzymes involved in the acquisition of N and P, such as the β-glucosaminidase phosphatase, could be used to infer microbial nutrient demand for N and P (Marklein and Houlton, 2012). Therefore, fertilisers-induced microbial response should be also assessed by soil enzyme activities (Hu et al., 2014; Rosinger et al., 2019). Thus, we measured the activities of soil urease, invertase, peroxidase, and acid phosphatase by using colorimetric method for three times, respectively (Guan, 1983).

### Data Analysis

All the response variables, including soil pH and nutrients, plant community characteristics, soil enzyme activities, and soil microbial indexes, along N and P addition rates, were analysed by the one-way ANOVA followed by Duncan’s multiple range test by using SPSS 19.0 software package (SPSS Incorporation, Chicago, Illinois, United States), respectively. The data of response variables were transformed with the natural logarithm to improve normality.

We used multiple regressions on distance matrices (MRM) in the *ecodist* package to estimate the importance of plant and soil factors on soil microbial PLFAs and enzyme activities. The respective soil microbial PLFAs were indicated by soil microbial PLFA biomass, PLFA richness, and the PLFA biomass of GB, the G^+^, the G^–^, Ac, and fungi. The plant factors included plant biomass, plant richness, root biomass, and the biomass of forbs, grasses, legumes, and sedges. The soil factors included soil pH, AN, AP, TN, TP, and SOM. The Euclidean distance matrices for the plant, soil factors, and microbial variables standardised with decostand function (standardise method) of the vegan package were used in MRM models. Then, the Spearman’s rank correlation test was to check the relationships between soil microbial variables and factors of the plant and soil.

Structural equation modeling (SEM) was used to analyse the hypothetical pathways of N and P addition effects on soil enzyme activities and soil microbial communities by using Amos version 23.0 (Amos Development, Spring House, Armonk, United States). Before the SEM analysis, the dominant factors affecting soil microbial variables according to the Spearman’s rank correlation test were selected. Soil microbial PLFAs were presented by the first principal components (PLFA PC1) and the second principal components (PLFA PC2). Soil enzyme activities were presented by the first principal components (enzyme PC1) and the second principal components (enzyme PC2) in the following SEM analysis. Maximum likelihood estimation method was applied in the process of SEM and the goodness-of-fit of the models was determined by the chi-squared tests, the Akaike information criterion (AIC), and the root mean square error of approximation (RMSEA).

## Results

### Soil Physicochemical Properties

**FIGURE 1:**
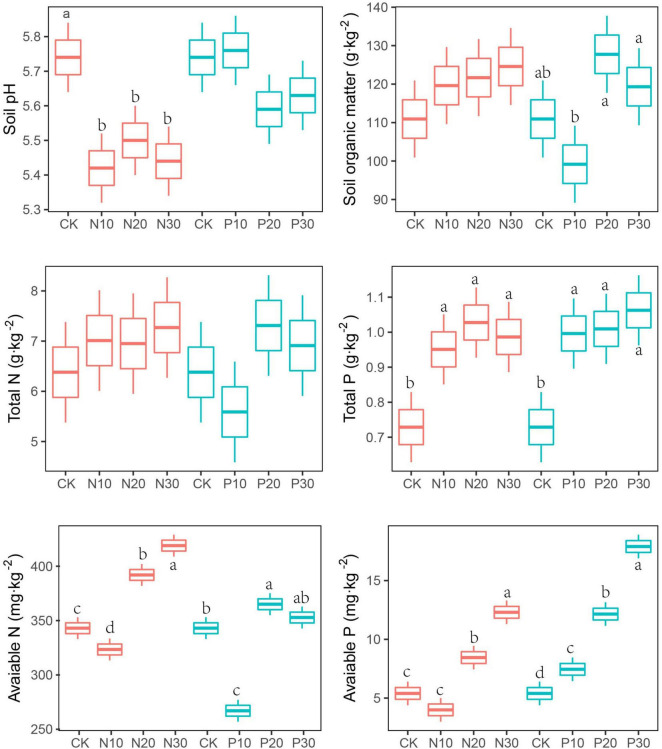
Effects of nitrogen (N) and phosphorus (P) addition on soil physicochemical. Values are means ± SE (*n* = 3). Different lowercase letters indicate significant difference among the levels of N or P addition in the same below.

The N addition significantly decreased soil pH, but not altered the contents of SOM and TN. Soil pH and TN were not significantly changed along P addition rates. Compared with unfertilised soils, the AN was lower in the low N and P addition rate (N10 and P10), but higher in the moderate and high addition rate (N20, P20, N30, and P30). The soil AP showed a significant increase with P addition rate (Figure 1 and Table 1).

**TABLE 1:** ANOVA for the four groups (soil physicochemical properties, plant community, soil enzyme activities, and soil microbial community) in nitrogen (N) and phosphorus (P) addition.

		N addition		P addition	
		F	*P*	F	*P*
Soil physicochemical properties	Soil pH	6.51	0.015	2.06	0.184
	Soil organic matter	1.03	0.426	4.47	0.040
	Total nitrogen	0.42	0.742	1.66	0.252
	Total phosphorus	5.39	0.025	6.66	0.014
	Available nitrogen	57.79	<0.001	58.64	<0.001
	Available phosphorus	40.59	<0.001	92.59	<0.001
Plant communities	Plant biomass	3.01	0.059	8.11	0.001
	Plant richness	5.33	0.009	2.51	0.093
	Grass biomass	102.85	<0.001	1.35	0.291
	Sedge biomass	6.24	0.005	3.47	0.039
	Legume biomass	9.14	0.001	10.79	<0.001
	Forbs biomass	4.24	0.021	4.28	0.020
	Root biomass	15.01	<0.001	13.89	<0.001
Soil enzyme activities	Urease	26.27	<0.001	9.79	0.001
	Invertase	11.28	<0.001	7.32	0.003
	Peroxidase	16.29	<0.001	17.09	<0.001
	Phosphatase	16.67	<0.001	13.42	<0.001
Soil microbial community	PLFA biomass	311.67	<0.001	141.97	<0.001
	PLFA richness	2.15	0.172	18.04	0.001
	General bacteria	805.76	<0.001	35.52	<0.001
	Fungi	4.85	0.033	45.30	<0.001
	Gramme-positive bacteria	84.80	<0.001	23.55	<0.001
	Gramme-negative bacteria	2.15	0.172	5.00	0.031
	Actinomycetes	4.06	0.050	3.99	0.052

### Plant Community

**FIGURE 2:**
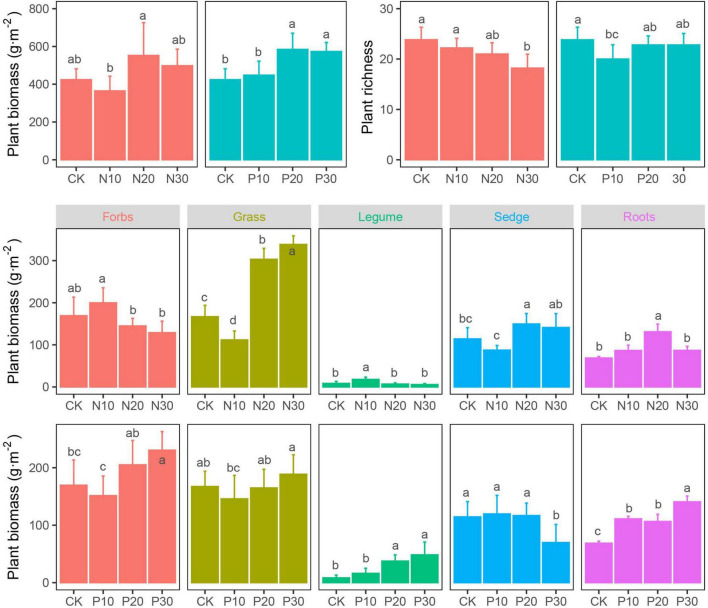
Effects of N and P addition on plant community characteristics.

The N addition did not significantly alter plant community biomass, which was significantly increased in P addition treatment, especially in moderate and high addition rate. Both the N and P addition decreased the plant richness (Figure 2 and Table 1).

Although the plant community biomass was not significantly induced by N addition, the response of four plant functional groups was significantly different. Low N addition rate (N10) increased forb and legume biomass, but inhibited grass and sedge biomass, which was opposite to the response of their biomass to N20 and N30 addition. The forb and grass biomass decreased in P10, but increased in P20 and P30, respectively. The legume biomass significantly increased with the P addition rate. The sedge biomass was not altered in P10 and P20, but significantly decreased in P30. Plant root biomass was increased following N and P addition rate, but its response was more sensitive in P addition than that in N addition, of which only N20 significantly increased root biomass (Figure 2 and Table 1).

### Soil Enzyme Activities

**FIGURE 3:**
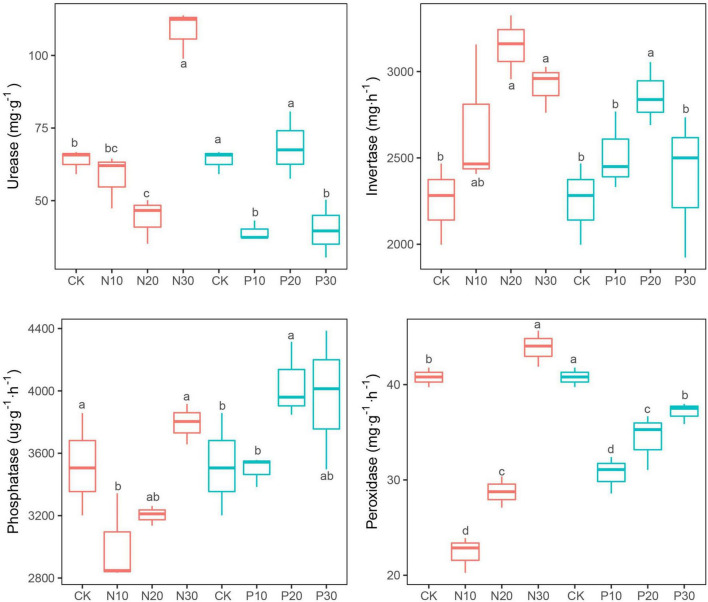
Effects of N and P addition on soil enzyme activities.

Invertase activities uniformly increased in response to the N and P addition and reached the maximum in N20 and P20 (Figure 3). The peroxidase, urease, and phosphatase activities significantly declined in N10 and N20, but were significantly increased in N30 (Figure 3). The response of these three soil enzyme activities varied in response to P addition. Peroxidase activity was significantly inhibited (Figure 3), while phosphatase activity increased with P addition (Figure 3). Urease activity was significantly decreased in P10 and P30, but slightly increased in P20 (Figure 3).

### Soil Microbial Composition

**FIGURE 4:**
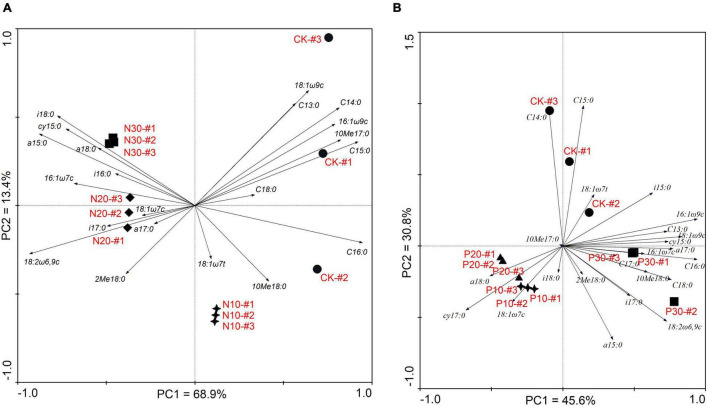
Soil microbial composition along N **(A)** and P **(B)** addition rates based on principal component analysis (PCA).

Principal component analysis of the soil microbial PLFA composition along N and P addition rate is shown in Figure 4. The PC1 explained 68.9% and the PC2 explained 13.4% of the variance for the N addition, respectively (Figure 4A). The locations of the three N addition rates and CK were distinct, which indicated that the dominant PLFA was different from each other. All the G^+^ indicated by PLFA a15:0, i18:0, a18:0, i17:0, a 17:0, and i16:0 were on the left of the PC2, while the GB indicated by PLFA C13:0, C14:0, C15:0, and C16:0 were on the right of the PC2. This demonstrated that along N addition rate, the G^+^ were increasing, especially in the high N addition rate, while fewer GB were found. Otherwise, the G^–^ indicated by PLFA cy 15:0, 16:1ω7c, 18:1ω7c, 18:1ω7t, and 16:1ω9c, Ac indicated by PLFA 2Me 18:0, 10Me 18:0, and 10Me 17:0, and fungal PLFA indicated by 18:1ω9c and 18:2ω6,9c were found along the PC1 axis from −1.0 to 1.0, which demonstrated that these soil PLFAs were not different between N addition and the CK.

The PC1 and PC2 explained the 45.6 and 30.8% of the variance for the P addition (Figure 4B). Although P addition differentiated the soil PLFAs from the CK, the PLFAs in low (P10) and moderate P addition rate (P20) were similar, in which both the G^+^ and the G^–^ PLFAs were found. It was distinct that the fungal PLFAs and Ac PLFAs were located near the high P addition rate (P30), while the whole GB PLFAs were found near the CK. These results demonstrated that soil microbial PLFAs were sensitive to P addition, especially at the P30 addition rate.

Although the N addition dominated the soil G^+^, while P addition affected soil fungi and Ac; some soil microorganisms at the individual PLFA level showed similar responses to N and P addition. These PLFAs, such as the saturated fatty acid C13:0, C14:0, and C15:0, as well as some monochain fatty acids 16:1ω9c and 18:1ω9c, were always located near the CK and not affected by the N or P addition. On the contrary, some PLFAs, such as a15:0 and 16:1ω7c, were found near the N30 and P30, and the PLFAs 18:1ω7c was found near the N20 and P20 (Figure 4).

### Soil Microbial Biomass

**FIGURE 5:**
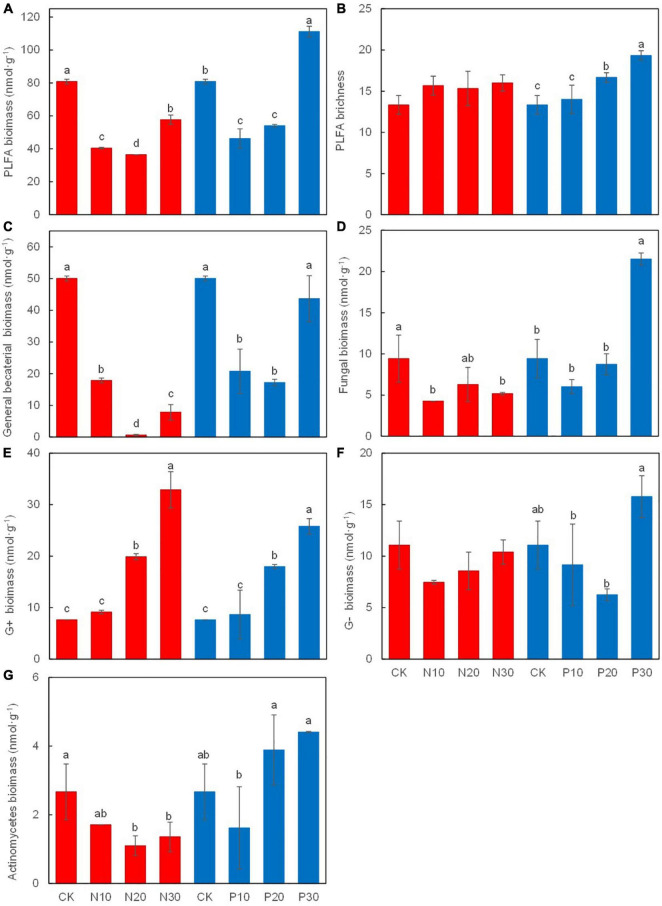
Effects of N and P addition on soil microbial biomass **(A)**, PLFA richness **(B)**, the biomass of grenral bacteria **(C)**, fungi **(D)**, gram-positive bacteria **(E)**, gram-negative bacteria **(F)**, and actinomycetes **(G)**.

Soil PLFA biomass (Figure 5A), including GB (Figure 5C), fungal (Figure 5D), and Ac biomass (Figure 5G), was significantly decreased along N addition rates, while the G^+^ were significantly increased (Figure 5E). Soil PLFA richness (Figure 5B) and the G^–^ (Figure 5F) were not statistically significant, while they showed an increasing and decreasing trend along N addition rates, respectively.

Along the P addition rates, the biomass of soil microorganisms, GB, fungi, and the G^–^ was first decreased and then increased at the high P addition rate (P30), which was significantly higher than that in the unfertilised treatment (CK). Soil PLFA richness and the G^+^ were significantly increased along the P addition rates (Figure 5).

### Importance of Plant and Soil Factors on Soil Microbial Variables

**FIGURE 6:**
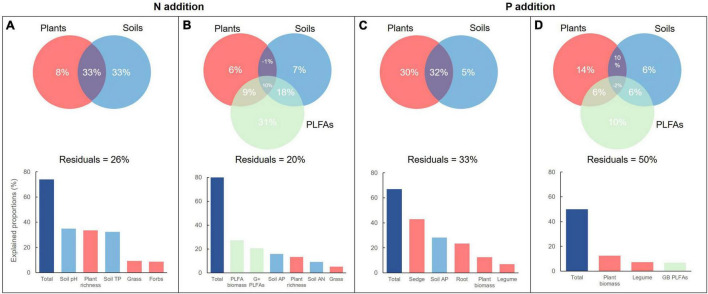
The relative contributions of plants and soils to the variations in soil PLFAs **(A,C)** and enzyme activities **(B,D)** along N and P addition rates.

Multiple regressions on distance matrices was used to estimate the importance of ecological factors (plant and soil factors) on soil microbial variables. Collectively, 74 and 67% of the total variations occur in soil microbial PLFAs of the N and P addition, respectively (Figures 6A,C). However, 8% of the total variations was alone explained by plant factors in the N addition, which was significantly lower than that (30%) in the P addition. On the contrary, the soil factors alone explained 33% in the N addition and 5% of the total variations in the P addition. This suggested that soil factors dominated soil microbial PLFAs in the N addition, but plant factors did not dominated soil microbial PLFAs in the P addition. Further investigation also showed that two most important variables were soil pH and plant richness in the addition, but the sedge biomass and soil AP in the P addition.

When the soil microbial PLFAs were taken into account to explain soil enzyme activities, the overall 80 and 50% of the total variations occur in the N and P addition, respectively (Figures 6B,D). Plants, soils, and PLFAs alone explained 6, 7, and 31% in the N addition, but 14, 6, and 10% of the total variations in the P addition. The biomass of total PLFAs and the G^+^ was the two most important variables explaining soil enzyme activities, but the biomass of total plants and legumes was the two important variables, followed by the GB PLFAs.

### Pathways Determining Soil Microbial Phospholipid Fatty Acids and Enzyme Activities

Based on the most important factors explaining soil microbial variables, we conducted an optimal SEM analysis to explore the pathways determining soil microbial variables. The results showed that the pathways of N and P addition determining soil microbial PLFAs and enzyme activities were significantly different.

**FIGURE 7:**
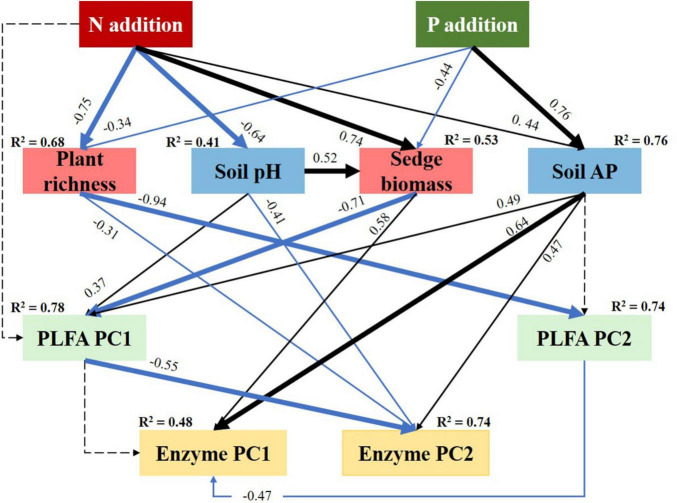
Structural equation modeling (SEM) analysis evaluating the effects of N and P addition on soil microbial PLFAs and enzyme activities via pathways of plants and soils. Result of model fitting: chi-squared test = 23.56, df = 21, and *P* = 0.315. Here, high *P*-values with the chi-squared tests indicate good model fit to data. Black and blue solid arrows indicate significantly positive and negative effects (*P* < 0.05), respectively. Dashed arrows indicate the effects are not significant (*P* > 0.05). Values associated with arrows represent standardised path coefficients. *R*^2^ associated with response variables indicates the proportion of variation explained by relationships with other variables.

The width of lines from N addition to the plant and soil factors showed that N addition would drive soil microbial variables mainly via decreasing plant richness (−0.75), increasing sedge biomass (0.74), followed by decreasing soil pH (−0.64) (Figure 7). The total effects of N addition on the soil microbial PLFA PC2 was 0.008 (Supplementary Figure 1A), but it reached 0.808 on soil microbial PLFA PC2 (Supplementary Figure 1C). Similarly, the total effects of N addition on soil enzyme PC2 were 0.690 (Supplementary Figure 1D), which were bigger than that (0.145) on soil enzyme PC1 (Supplementary Figure 1B).

The P addition would significantly affect soil microbial variables mainly via increasing AP (0.76) determined, followed by decreasing sedge biomass (−0.44) and plant richness (−0.34) (Figure 7). In addition, the total effects of P addition on soil microbial PLFA PC1 and enzyme PC1 were more than the corresponding PC2, respectively (Supplementary Figure 1).

According to the PCA analysis (Supplementary Figure 2A), soil PLFA PC1 was mainly indicated by soil PLFA biomass, the biomass of fungi, and Ac. Soil PLFA PC2 was mainly positively correlated with the G^+^ and PLFA richness and negatively correlated with GB. They indicated that N addition would dominant the structure of the G^+^ and GB, while P addition would determine soil PLFA biomass, the biomass of fungi, and Ac. Similarly, P addition was more important to soil phosphatase and peroxidase activity, while N addition was more important to soil invertase activity (Supplementary Figure 2B).

## Discussion

The alpine meadows in the Qinghai-Tibetan Plateau are strongly limited in terms of available N and P levels (Xu et al., 2015; He et al., 2016). Soil microorganisms are an important decomposer in alpine meadows and they are sensitive to changes in nutrient supply such as N or P addition. This study examined soil microbial PLFAs, including the biomass of soil GB, fungi, G^–^, and Ac, which were all rate dependently inhibited by N addition. Although they decreased at low and moderate P addition rate (<20 g m^–2^ year^–1^), soil microbial PLFAs significantly increased at high P addition rate (30 g m^–2^ year^–1^) and showed the unimodal changes responding to P addition. Both the N and P effects were rate dependent, which support our first hypothesis.

### Effects of Nitrogen and Phosphorus Addition on Soil Physicochemical Properties

The urea containing ammonium N released more hydrogen (H^+^) into the soils through bacterial nitrification, thus reducing soil pH. High N addition rate saturating the soil with N was also suggested to be an important reason for N-induced soil acidification (Lu et al., 2014), consistent with global observations (Tian and Niu, 2015). In contrast to the response of soil pH to N addition, soil pH was not significantly altered by P addition. This might be for that N uptakes were enhanced by P addition, resulting in less excess N in the soils, potentially delaying soil acidification (Mori et al., 2010; Chen et al., 2015; Li et al., 2016).

Nitrogen and P addition would enhance plant carbon assimilation and stimulated more litters into soils and, thus, increased SOM. Meanwhile, N and P addition would also stimulate N and P absorbed by plants and it resulted in less carbon into soils through roots. The trade-off in the contribution of litter carbon and root carbon into SOM was usually found in the low N and P addition rate and, thus, led to decrease in SOM. When high N and P addition rates were applied, litter carbon into soils surpassed root carbon inputs leading to increased SOM (Van der Heijden et al., 2008).

Soil N and P were mainly formed from deposition and fertiliser supply. The N and P addition directly increased the soil TN and TP contents (Cao et al., 2015; Du, 2017), especially the N addition significantly increased soil TP content for the N-limited plants that would not uptake soil P and resulted in the P accumulation (Song et al., 2019; Li et al., 2022). However, the soil AN was significantly decreased at low N and P addition rate, but it increased at medium and high N and P addition rate. This was for that low N addition rate. The AN from low N addition rate would stimulate N-limited plants absorbing the more AN from soils, which resulted in lower soil available content (Zhang et al., 2005). The P addition has been demonstrated to be significantly correlated with soil nitrogen cycle (Shi et al., 2021) and it would significantly promote the *nifH* gene expression and the biological nitrogen fixation rate (Chu et al., 2008).

### Effects of Nitrogen and Phosphorus Addition on the Plant Functional Groups

Previous studies found that lack of N and P availability can be limiting factors to plant growth and productivity in grasslands (Craine and Jackson, 2009; Fay et al., 2015), but in this study, the biomass of the four plant functional groups was differently responding to N and P addition. High N addition rate favoured grasses over the other functional groups, while P addition favoured the growth of legumes in the alpine meadow. The N-induced increase in grasses was consistent with most studies hypothesising that the plant community would be dominated by grasses in grassland ecosystems with N addition (Clark and Tilman, 2008; You et al., 2017; Zheng et al., 2019) due to their greater ability to take-up nutrients (Guo et al., 2017). The P-induced increase in legumes (Zhao et al., 2019) demonstrated that although the plant community was limited by N and P, the legumes were mostly limited by P due to their N-fixing ability (Marleau et al., 2011). Therefore, in an alpine meadow, N or P addition would drive the differentiation of plant community composition and structure (Tischer et al., 2015; Xu et al., 2015).

### Effects of Nitrogen and Phosphorus Addition on Soil Enzyme Activities

Soil enzymes are mainly produced by soil microorganisms to decompose organic matter into available nutrients for its assimilation and soil enzymes activities could be an indicator of soil microbial nutrient demand (Fan et al., 2018). N addition significantly increased the invertase activity (Figure 3), as found in other studies (Xiao et al., 2021). This result supports the resource allocation theory, which states that N addition stimulates the activities of C- and P-cycling enzymes, but inhibits N-cycling enzyme activities (Allison and Vitousek, 2005). However, we found that peroxidase activity indicative of C-cycling, urease activity indicative of N-cycling, and phosphatase activity indicative of P-cycling were first decreased at low N rates and then increased at high N rates, which were partially consistent with these results (Zhou et al., 2012; Han et al., 2018). The non-linear changes in soil enzyme activities are affected by a series of abiotic and biotic factors such as soil nutrient contents, soil microbial communities, soil respiration intensity, and plant community characteristics (Hu et al., 2014), especially in terms of soil microbial biomass (Acosta-Martínez et al., 2008). In this study, soil enzyme activities corresponding to N addition rates were mainly consistent with changes in soil PLFA biomass.

Our results showed that P addition generally suppressed peroxidase and urease activities, but increased invertase activities. Phosphatase activity only increased significantly at medium and high P addition rates, but showed no significant changes at low rate (Figure 3). These results contrasted with other studies (Jing et al., 2016; Rosinger et al., 2019; Xiao et al., 2021) showing negative effects of P addition on soil enzymes, especially soil phosphatase activity, where soil microorganisms would reduce the production cost of N-rich phosphatase enzymes when the available P was enough. In this study, although soil TP (1.01–1.06 g⋅kg^–1^) and AP (12.15–17.90 mg⋅kg^–1^) increased along with P addition rates, they were far less than the TP (2.30 g⋅kg^–1^) in the alpine meadows soils in the Qinghai-Tibet Plateau (Wang et al., 2013). When soil P supply is not enough, the negative effects of P addition on phosphatase activity are not triggered and are, thus, delayed (Olander and Vitousek, 2000; Weintraub and Schimel, 2005). Insufficient P supply, in turn, stimulates plant roots secretion of phosphatase (Tadano and Sakai, 1991), which was also found in this study, where root biomass significantly increased with P addition rates (Figure 2). Additionally, phosphatase activity upon P addition might be derived from stabilised extracellular enzymes, which are not sensitive to P addition, rather than actively synthesised enzymes (Turner and Joseph, 2013).

### Effects of Nitrogen and Phosphorus Addition on Soil Microbial Phospholipid Fatty Acids

N and P addition altered the ratio of N and P supply for plants and soil microorganisms and, thus, affected their relationships, growth, and biomass. Čapek et al. (2018) demonstrated that when the soil N-P ratio was between 6.3 and 42.4, plants were N limited and soil microorganisms were P limited. In this study, the soil N:P ratio at the three N and P addition rates was < 9.0 (Supplementary Figure 3), which further demonstrated that soil microorganisms in the grasslands were mainly limited by P (Rosinger et al., 2019). The negative relationship between N addition and soil microbial biomass (Leff et al., 2015) was also found in this study. In the short term, soil microorganisms compete for available N in the soil better than plants (Kuzyakov and Xu, 2013; Wild et al., 2018), which further leads to a lower soil C:N ratio and less tested fungi (Soares and Rousk, 2019). As a whole, N addition stimulated the P-limited effect in soil microorganisms (Li et al., 2016) and inhibited the soil microbial process in the alpine meadows, which was consistent with other studies (Zhou et al., 2017; Wang et al., 2018).

In contrast with expectations, a low P rate did not alleviate the limiting effect of P on soil microorganisms in this study. It has been reported that soil microorganisms in low-P soil are involved in efficient P cycling (Beauregard et al., 2010) and most of the available P was used to increase plant root biomass in legume plants through N_2_ fixation (Augusto et al., 2013), which was also demonstrated (Figure 2). Additionally, high P addition rate might limit N for microbial activity (Vitousek et al., 2010) including soil GB, fungi, the G^–^, the G^+^, and Ac (Figure 5).

### Importance of Plants and Soils for Soil Microorganisms

The growth strategies and environmental tolerance of soil microbial taxa are diverse (Schimel et al., 2007; Dong et al., 2020) and the response of soil microbial taxa to changes in plants and soils induced by N and P addition should be usually different.

The sustained decline in soil microbial biomass with N addition rate, expected for the soil G^+^, was mainly seen in the decreasing soil pH, followed by plant richness and soil TP. Soil bacteria are sensitive to decreased soil pH (Bååth and Anderson, 2003), which would lead to limited available calcium and magnesium (Lucas et al., 2011) as well as aluminium toxicity (Bowman et al., 2008), inhibiting soil bacterial community. In general, soil bacteria taxa have relatively narrow growth tolerances of soil pH (Rousk et al., 2010) and deviations of 1.5 pH units from the *in situ* pH of bacterial communities consistently reduce their activity by 50% (Fernandez-Calvino and Baath, 2010). However, soil fungi generally exhibit wider soil pH ranges for optimal growth than bacteria (Beales, 2004; Rousk et al., 2010). Therefore, soil bacteria decreased significantly, but the extent of soil fungi change was smaller.

Nitrogen addition also altered soil microbial PLFAs through plant richness, which was almost equal to the effect of soil pH in this study (Figure 6A). Plant effects, such as biomass (Reich et al., 2012), diversity, and composition (Lange et al., 2014), could also determine soil microbial growth and composition. More plant species supply more food resources and more host species supply more soil microorganisms by increasing the diversity of available nutrients pools (Klironomos et al., 2011). In some cases, plant richness determines soil microbial biomass (Zeng et al., 2016). N addition would change the C allocation of the aboveground plants and belowground roots and further lead to the priming effects of C deficiency on soil microorganisms (Silva-Sánchez et al., 2019). In this study, the biomass of grasses and forbs also accounted for 9.35 and 8.76% of the variation in soil microbial PLFAs, respectively. The priming effects of grass and forbs might account for the specific soil microbial taxa. However, the soil G^+^ significantly increased with decreasing soil pH induced by N addition rates. This was consistent with previous studies (Aciego Pietri and Brookes, 2009; Zhou et al., 2017), which have demonstrated that the soil G^+^ possessed specific mechanisms to survive in acidic environments (Cotter and Hill, 2003). Simonin et al. (2017) also considered the soil G^+^ as the K-strategist indicating soil microbial community resistance.

Compared with N addition, P addition effects on soil microorganisms were obviously unimodal. Except for the soil G^+^, the other microbial groups significantly decreased at P10 and P20 rates and increased at P30 rate. This soil microbial pattern along P addition rates mainly derived from plants (Yang et al., 2012; Rui et al., 2015; Wang et al., 2018), which accounted for 30% of the total variation, compared with the 5% derived from soils (Figure 6). The low P addition rate would not significantly change plant biomass and even decreased the biomass of forbs and grass, which led to less C substrate to soil microorganisms. Meanwhile, increased root biomass at high P addition rate directly enhanced the rate of C cycles to soil microorganisms (Jing et al., 2017).

The overall increase in soil nutrients affected specific PLFAs in both the N and P addition. In this study, 16:1ω7c and 18:1ω7c PLFAs were significantly increased by N and P addition rate and their correlation with soil TP, AP, and AN content has been already demonstrated (Dong et al., 2014; Chen et al., 2019; Hu et al., 2019). Conversely, some PLFAs, such as C13:0, C14:0, C15:0, 16:1ω9c, and 18:1ω9c, were not significantly changed by N and P addition. This difference might be due to the different microorganism growth strategies and the varying communication between them (Schimel et al., 2007). For instance, fungal PLFA 18:1ω9c was more resistant to changing microenvironments compared with bacteria (Wardle et al., 2003), but soil bacteria were more inhibited by N addition (Zhang et al., 2016). Furthermore, soil microorganisms produce more *cis*-structured PLFAs, such as 16:1ω7c and 18:1ω7c, with abundant available nutrients (Ludvigsen et al., 1999). These changes in individual PLFA affected by N and P addition would together help to structure the soil microbial taxa.

In this study, soil microbial community and enzyme activities are sensitive and significantly affected by the N and P addition rates. The N addition has negative effects on soil microbial community and enzyme activities along the addition rates. However, the effects of P addition are negative at low addition rates, but positive at high addition rates. Specifically, the soil microbial PLFA, bacteria, fungi, G^–^, and Ac are significantly inhibited along N addition rates and at low P addition rates. However, these PLFA groups are increased at high P addition rate. It is similar that both the N and P additions stimulate soil microbial richness and significantly increase the G^+^. Soil enzyme activities involved in C cycling are increased, but that involved in P cycling are inhibited at low addition rates and increased in high addition rates. The responses of urease activities are significantly different to N and P addition rates. These changes in soil microbial community are significantly correlated with soil pH with N addition rates, but with soil available P and sedge biomass with P addition rates. In the alpine meadow, both the N and P are the key limiting factors structuring soil microorganisms and 30 g⋅m^–2^ are better P addition rate for soil microbial community. Our studies indicated that soil microbial communities and enzyme activities are sensitive to short-term N and P addition and are also significantly influenced by their addition rates.

## Data Availability Statement

The raw data supporting the conclusions of this article will be made available by the authors, without undue reservation.

## Author Contributions

LH and CW contributed to the design of the study. LH organised the database. HZ performed the statistical analysis. LH and HZ wrote the first draft of the manuscript. All authors contributed to manuscript revision, read, and approved the submitted version.

## Conflict of Interest

The authors declare that the research was conducted in the absence of any commercial or financial relationships that could be construed as a potential conflict of interest.

## Publisher’s Note

All claims expressed in this article are solely those of the authors and do not necessarily represent those of their affiliated organizations, or those of the publisher, the editors and the reviewers. Any product that may be evaluated in this article, or claim that may be made by its manufacturer, is not guaranteed or endorsed by the publisher.

## References

[B1] Aciego Pietri J. C. Brookes P. C. (2009). Substrate inputs and pH as factors controlling microbial biomass, activity and community structure in an arable soil. *Soil Biol. Biochem.* 41 1396–1405. 10.1016/j.soilbio.2009.03.017

[B2] Acosta-Martínez V. Acosta-Mercado D. Sotomayor-Ramírez D. Cruz-Rodríguez L. (2008). Erratum to “Microbial communities and enzymatic activities under different management in semiarid soils. *Appl. Soil Ecol.* 39 358–358. 10.1016/j.apsoil.2008.01.004

[B3] Allison S. D. Vitousek P. M. (2005). Responses of extracellular enzymes to simple and complex nutrient inputs. *Soil Biol. Biochem.* 37 937–944. 10.1016/j.soilbio.2004.09.014

[B4] Augusto L. Delerue F. Gallet-Budynek A. Achat D. L. (2013). Global assessment of limitation to symbiotic nitrogen fixation by phosphorus availability in terrestrial ecosystems using a meta-analysis approach. *Glob. Biogeochem. Cycles* 27 804–815. 10.1002/gbc.20069

[B5] Bååth E. Anderson T. H. (2003). Comparison of soil fungal/bacterial ratios in a pH gradient using physiological and PLFA-based techniques. *Soil Biol. Biochem.* 35 955–963. 10.1016/s0038-0717(03)00154-8

[B6] Beales N. (2004). Adaptation of microorganisms to cold temperatures, weak acid preservatives, low pH, and osmotic stress: a review. *Compr. Rev. Food Sci. Food Saf.* 3 1–20. 10.1111/j.1541-4337.2004.tb00057.x 33430556

[B7] Beauregard M. S. Hamel C. Atul N. St-Arnaud M. (2010). Long-term phosphorus fertilization impacts soil fungal and bacterial diversity but not AM fungal community in alfalfa. *Microb. Ecol.* 59 379–389. 10.1007/s00248-009-9583-z 19756847

[B8] Berendsen R. L. Pieterse C. M. Bakker P. A. (2012). The rhizosphere microbiome and plant health. *Trends Plant Sci.* 17 478–486. 10.1016/j.tplants.2012.04.001 22564542

[B9] Bowman W. D. Cleveland C. C. Halada Å Hreško J. Baron J. S. (2008). Negative impact of nitrogen deposition on soil buffering capacity. *Nat. Geosci.* 1 767–770. 10.1038/ngeo339

[B10] Bremner J. M. (1960). Determination of nitrogen in soil by the Kjeldahl method. *J. Agr. Sci.* 55 11–33. 10.1017/s0021859600021572

[B11] Brundrett M. C. (2009). Mycorrhizal associations and other means of nutrition of vascular plants: understanding the global diversity of host plants by resolving conflicting information and developing reliable means of diagnosis. *Plant Soil* 320 37–77. 10.1007/s11104-008-9877-9

[B12] Camenzind T. Hättenschwiler S. Treseder K. K. Lehmann A. Rillig M. C. (2018). Nutrient limitation of soil microbial processes in tropical forests. *Ecol. Monogr.* 88 4–21. 10.1002/ecm.1279

[B13] Cao W. X. Li W. Li X. L. Xu C. L. Shi S. L. Han T. H. (2015). Effects of nitrogen fertilization on plant community structure and soil nutrient in alpine meadow steppe. *J. Desert Res*. 35 658–666. 10.7522/j.issn.1000-694X.2015.00043

[B14] Čapek P. Manzoni S. Kastovska E. Wild B. Diakova K. Barta J. (2018). A plant-microbe interaction framework explaining nutrient effects on primary production. *Nat. Ecol. Evol.* 2 1588–1596. 10.1038/s41559-018-0662-8 30201963

[B15] Chen D. M. Lan Z. C. Bai X. Grace J. B. Bai Y. F. (2013). Evidence that acidification-induced declines in plant diversity and productivity are mediated by changes in below-ground communities and soil properties in a semi-arid steppe. *J. Ecol.* 101, 1322–1334. 10.1111/1365-2745.12119

[B16] Chen F. Tan M. Yang Y. Ma J. Zhang S. Li G. (2015). The diversity changes of soil microbial communities stimulated by climate, soil type and vegetation type analyzed via a functional gene array. *World J. Microbiol. Biotechnol*. 31 1755–1763. 10.1007/s11274-015-1926-y 26296414

[B17] Chen X. Hao B. Jing X. He J.-S. Ma W. Zhu B. (2019). Minor responses of soil microbial biomass, community structure and enzyme activities to nitrogen and phosphorus addition in three grassland ecosystems. *Plant Soil* 444 21–37. 10.1007/s11104-019-04250-3

[B18] Chu H. Fujii T. Morimoto S. Lin X. Yagi K. (2008). Population size and specific nitrification potential of soil ammonia-oxidizing bacteria under long-term fertilizer management. *Soil Biol. Biochem*. 40 1960–1963. 10.1016/j.soilbio.2008.01.006

[B19] Chu H. Lin X. Fujii T. Morimoto S. Yagi K. Hu J. (2007). Soil microbial biomass, dehydrogenase activity, bacterial community structure in response to long-term fertilizer management. *Soil Biol. Biochem.* 39 2971–2976. 10.1016/j.soilbio.2007.05.031

[B20] Clark C. M. Tilman D. (2008). Loss of plant species after chronic low-level nitrogen deposition to prairie grasslands. *Nature* 451 712–715. 10.1038/nature06503 18256670

[B21] Cotter P. D. Hill C. (2003). Surviving the acid test: responses of gram-positive bacteria to low pH. *Microbiol. Mol. Biol. Rev.* 67 429–453. 10.1128/MMBR.67.3.429-453.2003 12966143 PMC193868

[B22] Craine J. M. Jackson R. D. (2009). Plant nitrogen and phosphorus limitation in 98 North American grassland soils. *Plant Soil*. 334 73–84. 10.1007/s11104-009-0237-1

[B23] Cusack D. F. Silver W. L. Torn M. S. Burton S. D. Firestone M. K. (2011). Changes in microbial community characteristics and soil organic matter with nitrogen additions in two tropical forests. *Ecology* 92 621–632. 10.1890/10-0459.1 21608471

[B24] Dong J. Wang S. Niu H. Cui X. Li L. Pang Z. (2020). Responses of soil microbes and their interactions with plant community after nitrogen and phosphorus addition in a Tibetan alpine steppe. *J. Soils Sediments* 20 2236–2247. 10.1007/s11368-020-02586-3

[B25] Diáková K. Biasi C. Èapek P. Martikainen P. J. Marushchak M. E. Patova E. N. (2018). Variation in N2 fixation in subarctic tundra in relation to landscape position and nitrogen pools and fluxes. *Arct. Antarct. Alp. Res.* 48 111–125. 10.1657/aaar0014-064

[B26] Dong W. Y. Zhang X. Y. Dai X. Q. Fu X. L. Yang F. T. Liu X. Y. (2014). Changes in soil microbial community composition in response to fertilization of paddy soils in subtropical China. *Appl. Soil Ecol.* 84 140–147. 10.1016/j.apsoil.2014.06.007

[B27] Du Q. F. (2017). *The Responses of Degraded Typical Steppe Vegetation and Soil to Nitrogen and Phosphorus Fertilizations in Inner Mongolia.* Ph.D thesis, Pune: Southwest University.

[B28] Eisenhauer N. Bessler H. Engels C. Gleixner G. Habekost M. Milcu A. (2010). Plant diversity effects on soil microorganisms support the singular hypothesis. *Ecology* 91 485–496. 10.1890/08-2338.1 20392013

[B29] Fan Z. Wang X. Wang C. Bai E. (2018). Effect of nitrogen and phosphorus addition on soil enzyme activities: a meta-analysis. *Chinese J. Appl. Ecol.* 29 1266–1272. 10.13287/j.1001-9332.201804.024 29726237

[B30] Fay P. A. Prober S. M. Harpole W. S. Knops J. M. Bakker J. D. Borer E. T. (2015). Grassland productivity limited by multiple nutrients. *Nat. Plants* 1:15080. 10.1038/nplants.2015.80 27250253

[B31] Fernandez-Calvino D. Baath E. (2010). Growth response of the bacterial community to pH in soils differing in pH. *FEMS Microbiol. Ecol.* 73 149–156. 10.1111/j.1574-6941.2010.00873.x 20455934

[B32] Fierer N. Lauber C. L. Ramirez K. S. Zaneveld J. Bradford M. A. Knight R. (2012). Comparative metagenomic, phylogenetic and physiological analyses of soil microbial communities across nitrogen gradients. *ISME J.* 6 1007–1017. 10.1038/ismej.2011.159 22134642 PMC3329107

[B33] Frostegård Å Bååth E. Tunlio A. J. (1993). Shifts in the structure of soil microbial communities in limed forests as revealed by phospholipid fatty acid analysis. *Soil Biol. Biochem.* 25 723–730. 10.1016/0038-0717(93)90113-p

[B34] Frostegård Å Bååth E. J. B. (1996). The use of phospholipid fatty acid analysis to estimate bacterial and fungal biomass in soil. *Biol. Fertil. Soils* 22 59–65. 10.1007/bf00384433

[B35] Frostegård Å Tunlid A. Bååth E. (2011). Use and misuse of PLFA measurements in soils. *Soil Biol. Biochem.* 43 1621–1625. 10.1016/j.soilbio.2010.11.021

[B36] Geisseler D. Scow K. M. (2014). Long-term effects of mineral fertilizers on soil microorganisms - A review. *Soil Biol. Biochem.* 75 54–63. 10.1016/j.soilbio.2014.03.023

[B37] Guan S. (1983). *Research Methods on Soil Enzymes.* Beijing: China Agricultural Press.

[B38] Guo H. Ye C. Zhang H. Pan S. Ji Y. Li Z. (2017). Long-term nitrogen & phosphorus additions reduce soil microbial respiration but increase its temperature sensitivity in a Tibetan alpine meadow. *Soil Biol. Biochem*. 113 26–34. 10.1016/j.soilbio.2017.05.024

[B39] Han Y. Liu Y. Ye Y. Da B. Gao Y. Zhao Y. (2018). The Effect of nitrogen deposition on soil enzyme activity in the forest-grassland landscape boundary of Tibet. *J. Land Res.* 10, 93-98.

[B40] Hartmann A. Schmid M. Tuinen D. Berg G. (2008). Plant-driven selection of microbes. *Plant Soil* 321 235–257. 10.1007/s11104-008-9814-y

[B41] He D. Xiang X. J. He J. S. Wang C. Cao G. M. Adams J. (2016). Composition of the soil fungal community is more sensitive to phosphorus than nitrogen addition in the alpine meadow on the Qinghai-Tibetan Plateau. *Biol. Fertil. Soils* 52 1059–1072. 10.1007/s00374-016-1142-4

[B42] Hu K. Tao J. He D. Huang K. Wang W. (2019). Effects of root growth on dynamic of microbes and enzyme activities during litter decomposition. *Chinese J. Appl. Ecol.* 30 1993–2001. 10.13287/j.1001-9332.201906.022 31257772

[B43] Hu L. Wang C. Wang G. Ma L. Liu W. Xiang Z. (2014). Changes in the activities of soil enzymes and microbial community structure at different degradation successional stages of alpine meadow in the headwater region of Three Rivers. *China*. *Acta Prataculturae Sin.* 23 8–19. 10.11686/cyxb20140302

[B44] Hu L. Zi H. Ade L. Lerdau M. Wang C. (2017). Effects of zokors (*Myospalax baileyi*) on plant, on abiotic and biotic soil characteristic of an alpine meadow. *Ecol. Eng.* 103 95–105. 10.1016/j.ecoleng.2017.03.010

[B45] Humbert J. Y. Dwyer J. M. Andrey A. Arlettaz R. (2016). Impacts of nitrogen addition on plant biodiversity in mountain grasslands depend on dose, application duration and climate: a systematic review. *Glob. Chang. Biol.* 22 110–120. 10.1111/gcb.12986 26010833

[B46] Isbell F. Reich P. B. Tilman D. Hobbie S. E. Polasky S. Binder S. (2013). Nutrient enrichment, biodiversity loss, and consequent declines in ecosystem productivity. *Proc. Nat. Acad. Sci.* 110 11911–11916. 10.1073/pnas.1310880110 23818582 PMC3718098

[B47] Jackson M. (1974). *Soil Chemical Analysis – An Advanced Course.* Madison, WI: Department of Soil Science, University of Wisconsin.

[B48] Jing D. W. Liu F. C. Wang M. Y. Ma H. L. Du Z. Y. Ma B. Y. (2017). Effects of root pruning on the physicochemical properties and microbial activities of poplar rhizosphere soil. *PLoS One* 12:e0187685. 10.1371/journal.pone.0187685 29117215 PMC5678864

[B49] Jing X. Yang X. Ren F. Zhou H. Zhu B. He J. S. (2016). Neutral effect of nitrogen addition and negative effect of phosphorus addition on topsoil extracellular enzymatic activities in an alpine grassland ecosystem. *Appl. Soil Ecol.* 107 205–213. 10.1016/j.apsoil.2016.06.004

[B50] Klironomos J. Zobel M. Tibbett M. Stock W. D. Rillig M. C. Parrent J. L. (2011). Forces that structure plant communities: quantifying the importance of the mycorrhizal symbiosis. *New Phytol.* 189 366–370. 10.1111/j.1469-8137.2010.03550.x 21058952

[B51] Kuzyakov Y. Xu X. (2013). Competition between roots and microorganisms for nitrogen: mechanisms and ecological relevance. *New Phytol.* 198 656–669. 10.1111/nph.12235 23521345

[B52] Lagrange A. L’Huillier L. Amir H. (2013). Mycorrhizal status of Cyperaceae from New Caledonian ultramafic soils: effects of phosphorus availability on arbuscular mycorrhizal colonization of *Costularia comosa* under field conditions. *Mycorrhiza* 23 655–661. 10.1007/s00572-013-0503-1 23636807

[B53] Lange M. Habekost M. Eisenhauer N. Roscher C. Bessler H. Engels C. (2014). Biotic and Abiotic properties mediating plant diversity effects on soil microbial communities in an experimental grassland. *PLoS One* 9:e96182. 10.1371/journal.pone.0096182 24816860 PMC4015938

[B54] Leff J. W. Jones S. E. Prober S. M. Barberan A. Borer E. T. Firn J. L. (2015). Consistent responses of soil microbial communities to elevated nutrient inputs in grasslands across the globe. *Proc. Natl. Acad. Sci. U.S.A.* 112 10967–10972. 10.1073/pnas.1508382112 26283343 PMC4568213

[B55] Li C. Y. Miao Y. Xue Y. L. Zhang B. B. Wang Y. Dang Y. H. (2022). Ecological stoichiometric characteristics of soil-microorganism-plant system in the Loess upland under long-term fertilization. *Acta Ecol. Sinica* 42 1–9. 10.5864/stxb202003050411

[B56] Li Y. Niu S. Yu G. (2016). Aggravated phosphorus limitation on biomass production under increasing nitrogen loading: a meta-analysis. *Glob. Chang. Biol*. 22 934–943. 10.1111/gcb.13125 26463578

[B57] Liu Y. Z. Zhao C. C. Guo J. W. Zhang L. N. Juan X. Chen A. Q. (2020). Short-term phosphorus addition augments the effects of nitrogen addition on soil respiration in a typical steppe. *Sci. Total Environ.* 761:143211. 10.1016/j.scitotenv.2020.143211 33172642

[B58] Lu X. Mao Q. Gilliam F. S. Luo Y. Mo J. (2014). Nitrogen deposition contributes to soil acidification in tropical ecosystems. *Glob. Chang. Biol*. 20 3790–3801. 10.1111/gcb.12665 24953639

[B59] Lucas R. W. Klaminder J. Futter M. N. Bishop K. H. Egnell G. Laudon H. (2011). A meta-analysis of the effects of nitrogen additions on base cations: implications for plants, soils, and streams. *For. Ecol. Manage.* 262 95–104. 10.1016/j.foreco.2011.03.018

[B60] Ludvigsen L. Albrechtsen H. Ringelberg D. B. Ekelund F. Christensen T. H. (1999). Distribution and composition of microbial populations in a landfill leachate contaminated aquifer (Grindsted, Denmark). *Microb. Ecol.* 37 197–207. 10.1007/s002489900143 10227877

[B61] Mao Q. Lu X. Zhou K. Chen H. Zhu X. Mori T. (2017). Effects of long-term nitrogen and phosphorus additions on soil acidification in an N-rich tropical forest. *Geoderma* 285 57–63. 10.1016/j.geoderma.2016.09.017

[B62] Marklein A. R. Houlton B. Z. (2012). Nitrogen inputs accelerate phosphorus cycling rates across a wide variety of terrestrial ecosystems. *New Phytol.* 193 696–704. 10.1111/j.1469-8137.2011.03967.x 22122515

[B63] Marleau J. N. Jin Y. Bishop J. G. Fagan W. F. Lewis M. A. (2011). A stoichiometric model of early plant primary succession. *Am. Nat.* 177 233–245. 10.1086/658066 21460559

[B64] McKinley V. L. Peacock A. D. White D. C. (2005). Microbial community PLFA and PHB responses to ecosystem restoration in tallgrass prairie soils. *Soil Biol. Biochem.* 37 1946–1958. 10.1016/j.soilbio.2005.02.033

[B65] Mori T. Ohta S. Ishizuka S. Konda R. Wicaksono A. Heriyanto J. (2010). Effects of phosphorus addition on N2O and NO emissions from soils of anAcacia mangiumplantation. *Soil Sci. Plant Nutr*. 56 782–788. 10.1111/j.1747-0765.2010.00501.x

[B66] Navratilova D. Tlaskalova P. Kohout P. Drevojan P. Fajmon K. Chytry M. (2019). Diversity of fungi and bacteria in species-rich grasslands increases with plant diversity in shoots but not in roots and soil. *FEMS Microbiol. Ecol.* 95:fiy208. 10.1093/femsec/fiy208 30312395

[B67] Olander L. P. Vitousek P. M. (2000). Regulation of soil phosphatase and chitinase activity by N and P availability. *Biogeochemistry* 49 175–191. 10.1023/a:1006316117817

[B68] Porazinska D. L. Farrer E. C. Spasojevic M. J. Bueno de Mesquita C. P. Sartwell S. A. Smith J. G. (2018). Plant diversity and density predict belowground diversity and function in an early successional alpine ecosystem. *Ecology* 99 1942–1952. 10.1002/ecy.2420 30024640

[B69] Ramsey P. W. Rillig M. C. Feris K. P. Holben W. E. Gannon J. E. (2006). Choice of methods for soil microbial community analysis: PLFA maximizes power compared to CLPP and PCR-based approaches. *Pedobiologia* 50 275–280. 10.1016/j.pedobi.2006.03.003

[B70] Reich P. B. Tilman D. Isbell F. Mueller K. Hobbie S. E. Flynn D. F. (2012). Impacts of biodiversity loss escalate through time as redundancy fades. *Science* 336 589–592. 10.1126/science.1217909 22556253

[B71] Ren F. Song W. Chen L. Mi Z. Zhang Z. Zhu W. (2016). Phosphorus does not alleviate the negative effect of nitrogen enrichment on legume performance in an alpine grassland. *J. Plant Ecol.* 10 822–830. 10.1093/jpe/rtw089

[B72] Rosinger C. Rousk J. Sanden H. (2019). Can enzymatic stoichiometry be used to determine growth-limiting nutrients for microorganisms? A critical assessment in two subtropical soils. *Soil Biol. Biochem.* 128 115–126. 10.1016/j.soilbio.2018.10.011

[B73] Rui J. Li J. Wang S. An J. Liu W. T. Lin Q. (2015). Responses of bacterial communities to simulated climate changes in alpine meadow soil of the Qinghai-Tibet Plateau. *Appl. Environ. Microbiol.* 81 6070–6077. 10.1128/AEM.00557-15 26116682 PMC4551261

[B74] Rousk J. Baath E. Brookes P. C. Lauber C. L. Lozupone C. Caporaso J. G. (2010). Soil bacterial and fungal communities across a pH gradient in an arable soil. *ISME J.* 4 1340–1351. 10.1038/ismej.2010.58 20445636

[B75] Schimel J. Balser T. Wallenstein M. (2007). Microbial stress-response physiology and its implications for ecosystem function. *Ecology* 88 1386–1394. 10.1890/06-0219 17601131

[B76] Shi W. Zhang L. M. Wang J. S. Li C. P. Gu Z. L. Zhao H. Y. (2021). The subsequent effects of phosphorus fertilization in upland red soils and the underlying mechanisms. *Acta Pedologica Sin.* 1–14.

[B77] Silva-Sánchez A. Soares M. Rousk J. (2019). Testing the dependence of microbial growth and carbon use efficiency on nitrogen availability, pH, and organic matter quality. *Soil Biol. Biochem.* 134 25–35. 10.1016/j.soilbio.2019.03.008

[B78] Simonin M. Nunan N. Bloor J. M. G. Pouteau V. Niboyet A. (2017). Short-term responses and resistance of soil microbial community structure to elevated CO_2_ and N addition in grassland mesocosms. *FEMS Microbiol. Lett.* 364:fnx077. 10.1093/femsle/fnx077 28430942

[B79] Soares M. Rousk J. (2019). Microbial growth and carbon use efficiency in soil: links to fungal-bacterial dominance, SOC-quality and stoichiometry. *Soil Biol. Biochem.* 131 195–205. 10.1016/j.soilbio.2019.01.010

[B80] Song Y. H. Ai Z. M. Qiao L. L. Zhai J. Y. Li Y. Z. Li Y. Y. (2019). Effects of fertilization on ecological stoichiometric ratio soil carbon, nitrogen, and phosphorus in farmland of the Loess Plateau. *Res. Soil Water Conser.* 26 38–45. 10.13869/j.cnki.rswc.2019.06.006

[B81] Tadano T. Sakai H. (1991). Secretion of acid phosphatase by the roots of several crop species under phosphorus-deficient conditions. *Soil Sci. Plant Nutr.* 37 129–140. 10.1080/00380768.1991.10415018

[B82] Tian D. Niu S. (2015). A global analysis of soil acidification caused by nitrogen addition. *Environ. Res. Lett*. 10:024019. 10.1088/1748-9326/10/2/024019

[B83] Tischer A. Werisch M. Dobbelin F. Camenzind T. Rillig M. C. Potthast K. (2015). Above- and belowground linkages of a nitrogen and phosphorus co-limited tropicalmountain pasture system-responses to nutrient enrichment. *Plant Soil* 391 333–352. 10.1007/s11104-015-2431-7

[B84] Treseder K. K. (2004). A meta-analysis of mycorrhizal responses to nitrogen, phosphorus, and atmospheric CO_2_ in field studies. *New Phytol.* 164 347–355. 10.1111/j.1469-8137.2004.01159.x 33873547

[B85] Treseder K. K. (2008). Nitrogen additions and microbial biomass: a meta-analysis of ecosystem studies. *Ecol. Lett.* 11 1111–1120. 10.1111/j.1461-0248.2008.01230.x 18673384

[B86] Turner B. L. Joseph W. S. (2013). The response of microbial biomass and hydrolytic enzymes to a decade of nitrogen, phosphorus, and potassium addition in a lowland tropical rain forest. *Biogeochemistry* 117 115–130. 10.1007/s10533-013-9848-y

[B87] Van der Heijden M. G. Bardgett R. D. van Straalen N. M. (2008). The unseen majority: soil microbes as drivers of plant diversity and productivity in terrestrial ecosystems. *Ecol. Lett*. 11 296–310. 10.1111/j.1461-0248.2007.01139.x 18047587

[B88] Vitousek P. M. Porder S. Houlton B. Z. Chadwick O. A. (2010). Terrestrial phosphorus limitation: mechanisms, implications, and nitrogen-phosphorus interactions. *Ecol. Appl.* 20 5–15. 10.1890/08-0127.1 20349827

[B89] Walkley A. Black I. A. (1934). An examination of the Degtjareff method for determining soil organic matter, and a proposed modification of the chromic acid Titration method. *Soil Sci.* 37 29–38. 10.1097/00010694-193401000-00003

[B90] Wang C. Cao Z. Wang L. Liu X. (2013). Ecological effects of leguminous plants on microorganism community in rhizosphere soils. *Ecol. Environ. Sci.* 22 85–89. 10.16258/j.cnki.1674-5906.2013.01.019

[B91] Wang C. Liu D. Bai E. (2018). Decreasing soil microbial diversity is associated with decreasing microbial biomass under nitrogen addition. *Soil Biol. Biochem.* 120 126–133. 10.1016/j.soilbio.2018.02.003

[B92] Wang J. Song B. Ma F. Tian D. Li Y. Yan T. (2019). Nitrogen addition reduces soil respiration but increases the relative contribution of heterotrophic component in an alpine meadow. *Funct. Ecol.* 33 2239–2253. 10.1111/1365-2435.13433

[B93] Wang Y. Li C. Kou Y. Wang J. Bo T. Li H. (2017). Soil pH is a major driver of soil diazotrophic community assembly in Qinghai-Tibet alpine meadows. *Soil Biol. Biochem.* 115 547–555. 10.1016/j.soilbio.2017.09.024

[B94] Wardle D. A. Nilsson M.-C. Zackrisson O. Gallet C. (2003). Determinants of litter mixing effects in a Swedish boreal forest. *Soil Biol. Biochem.* 35 827–835. 10.1016/s0038-0717(03)00118-4

[B95] Weintraub M. N. Schimel J. P. (2005). The seasonal dynamics of amino acids and other nutrients in Alaskan Arctic tundra soils. *Biogeochemistry* 73 359–380. 10.1007/s10533-004-0363-z

[B96] Wild B. Alves R. J. E. Bárta J. Èapek P. Gentsch N. Guggenberger G. (2018). Amino acid production exceeds plant nitrogen demand in Siberian tundra. *Environ. Res. Lett.* 13:034002. 10.1088/1748-9326/aaa4fa

[B97] Xiao H. Yang H. Zhao M. Monaco T. A. Rong Y. Huang D. (2021). Soil extracellular enzyme activities and the abundance of nitrogen-cycling functional genes responded more to N addition than P addition in an Inner Mongolian meadow steppe. *Sci. Total. Environ.* 759:143541. 10.1016/j.scitotenv.2020.143541 33198996

[B98] Xiao W. Chen X. Jing X. Zhu B. (2018). A meta-analysis of soil extracellular enzyme activities in response to global change. *Soil Biol. Biochem*. 123 21–32. 10.1016/j.soilbio.2018.05.001

[B99] Xu D. H. Fang X. W. Zhang R. Y. Gao T. P. Bu H. Y. Du G. Z. (2015). Influences of nitrogen, phosphorus and silicon addition on plant productivity and species richness in an alpine meadow. *AoB Plants* 7:plv125. 10.1093/aobpla/plv125 26574603 PMC4676797

[B200] Yang H. Jiang L. Li L. Li A. Wu M. Wan S. (2012). Diversity-dependent stability under mowing and nutrient addition: evidence from a 7-year grassland experiment. *Ecol. Lett.* 15 619–626. 10.1111/j.1461-0248.2012.01778.x 22487498

[B100] Yao M. Rui J. Li J. Dai Y. Bai Y. Hedìnec P. (2014). Rate-specific responses of prokaryotic diversity and structure to nitrogen deposition in the Leymus chinensis steppe. *Soil Biol. Biochem.* 79 81–90. 10.1016/j.soilbio.2014.09.009

[B101] You C. M. Wu F. Z. Gan Y. M. Yang W. Q. Hu Z. M. Xu Z. F. (2017). Grass and forbs respond differently to nitrogen addition: a meta-analysis of global grassland ecosystems. *Sci. Rep.* 7:1563. 10.1038/s41598-017-01728-x 28484219 PMC5431500

[B102] Zak D. R. Holmes W. E. White D. C. Peacock A. D. Tilman D. (2003). Plant diversity, soil microbial communities, and ecosystem function: are there any links? *Ecology* 84 2042–2050. 10.1890/02-0433

[B103] Zeng J. Liu X. Song L. Lin X. Chu H. (2016). Nitrogen fertilization directly affects soil bacterial diversity and indirectly affects bacterial community composition. *Soil Biol. Biochem.* 92 41–49. 10.1016/j.soilbio.2015.09.018

[B104] Zhang N. Wan S. Li L. Bi J. Zhao M. Ma K. (2008). Impacts of urea N addition on soil microbial community in a semi-arid temperate steppe in northern China. *Plant Soil* 311 19–28. 10.1007/s11104-008-9650-0

[B105] Zhang Q. Xie J. Lyu M. Xiong D. Wang J. Chen Y. (2016). Short-term effects of soil warming and nitrogen addition on the N:P stoichiometry of *Cunninghamia lanceolata* in subtropical regions. *Plant Soil* 411 395–407. 10.1007/s11104-016-3037-4

[B106] Zhang Y. D. Sun Z. H. Shen Y. X. (2005). Effect of fertilization on soil microorganism of deteriorated grassland in dry-hot valley region of Jinsha River. *J. Soil Water Conser*. 19 88–91. 10.13870/j.cnki.stbcxb.2005.02.03

[B107] Zhao Y. Yang B. Li M. Xiao R. Rao K. Wang J. (2019). Community composition, structure and productivity in response to nitrogen and phosphorus additions in a temperate meadow. *Sci. Total Environ.* 654 863–871. 10.1016/j.scitotenv.2018.11.155 30448675

[B108] Zheng Y. Kim Y. C. Tian X. F. Chen L. Yang W. Gao C. (2014). Differential responses of arbuscular mycorrhizal fungi to nitrogen addition in a near pristine Tibetan alpine meadow. *FEMS Microbiol. Ecol.* 89 594–605. 10.1111/1574-6941.12361 24890754

[B109] Zheng Z. Bai W. Zhang W. (2019). Root trait-mediated belowground competition and community composition of a temperate steppe under nitrogen enrichment. *Plant Soil* 437 341–354. 10.1007/s11104-019-03989-z

[B110] Zhou J. Jiang X. Zhou B. Zhao B. Ma M. Guan D. (2016). Thirty four years of nitrogen fertilization decreases fungal diversity and alters fungal community composition in black soil in northeast China. *Soil Biol. Biochem.* 95 135–143. 10.1016/j.soilbio.2015.12.012

[B111] Zhou T. C. Sun J. Shi P. L. (2021). Plant-microbe interactions regulate the aboveground community nitrogen accumulation rate in different environmental conditions on the Tibetan Plateau. *Catena* 204:105407. 10.1016/j.catena.2021.105407

[B112] Zhou X. Zhang Y. Downing A. (2012). Non-linear response of microbial activity across a gradient of nitrogen addition to a soil from the Gurbantunggut Desert, northwestern China. *Soil Biol. Biochem.* 47 67–77. 10.1016/j.soilbio.2011.05.012

[B113] Zhou Z. Wang C. Zheng M. Jiang L. Luo Y. (2017). Patterns and mechanisms of responses by soil microbial communities to nitrogen addition. *Soil Biol. Biochem.* 115 433–441. 10.1016/j.soilbio.2017.09.015

