# Supplementary figures and images for: Differentiate Responses of Soil Microbial Community and Enzyme Activities to Nitrogen and Phosphorus Addition Rates in an Alpine Meadow
Source: Front Plant Sci. 2022 Mar 2;13:829381. doi: 10.3389/fpls.2022.829381 (PMC8924503; doi:10.3389/fpls.2022.829381)

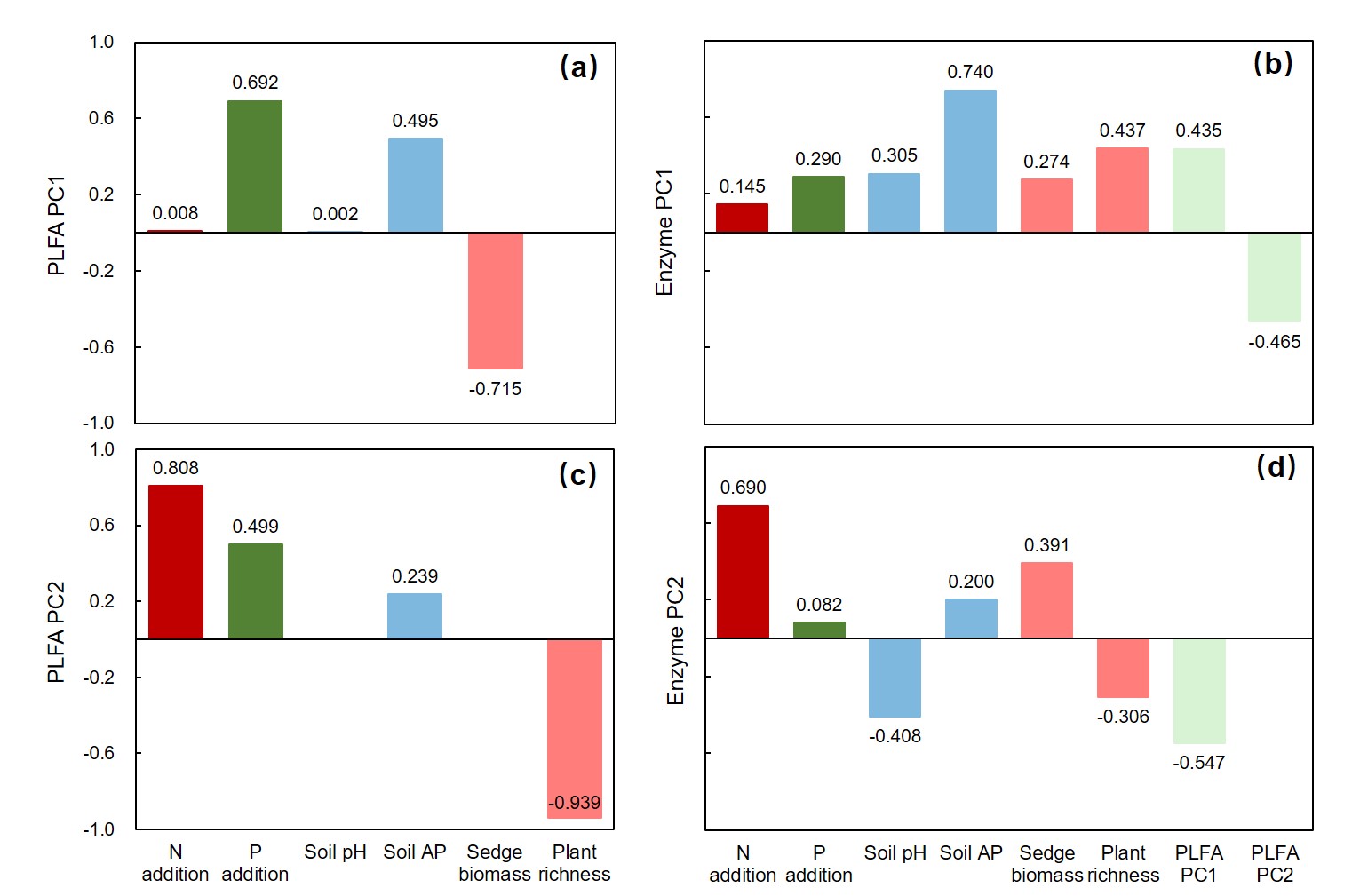

Supplement: Supplementary file 1 [file Image_1.jpeg]

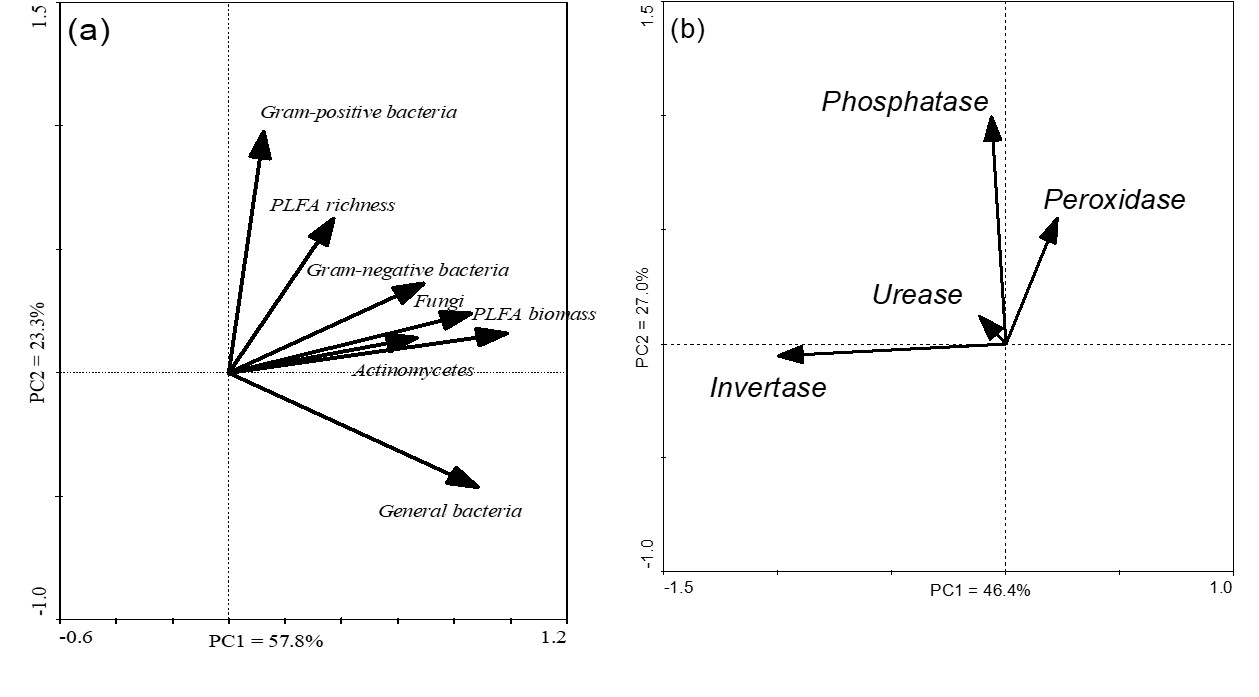

Supplement: Supplementary file 2 [file Image_2.jpeg]

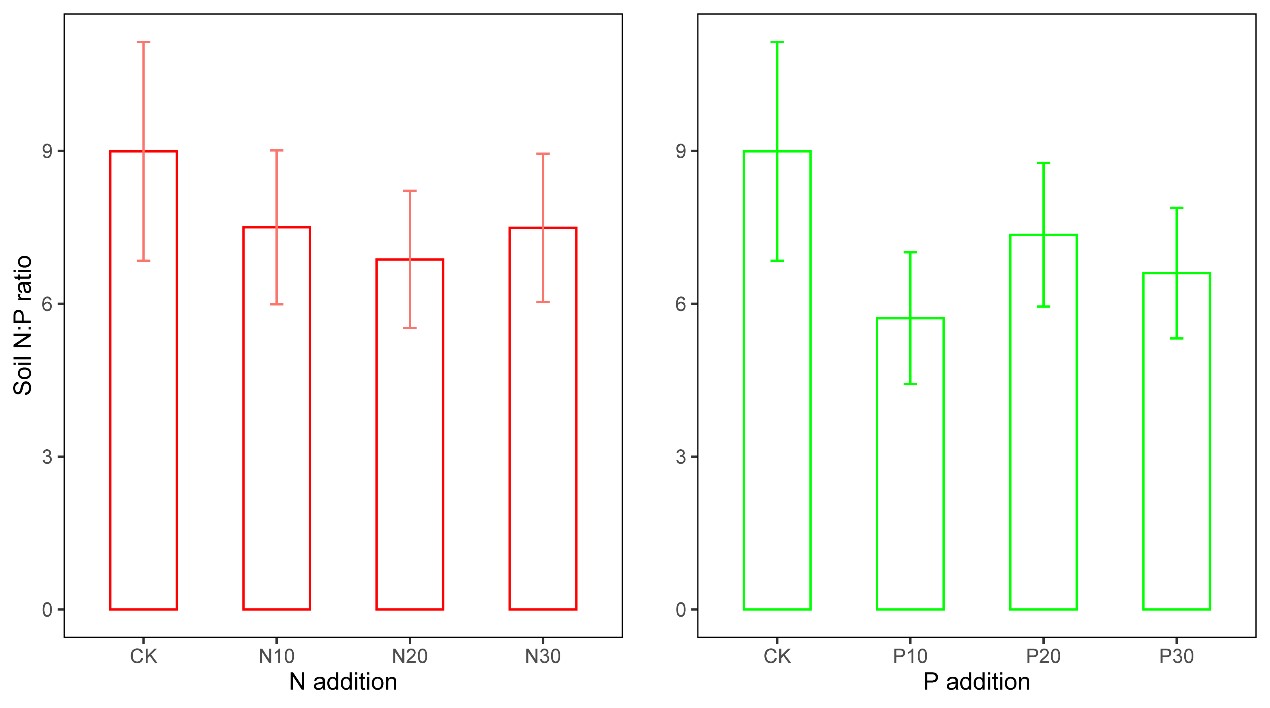

Supplement: Supplementary file 3 [file Image_3.jpeg]
